# Characterization of *Clostridium perfringens* Phage Endolysin PlyDolk21

**DOI:** 10.3390/antibiotics14010081

**Published:** 2025-01-13

**Authors:** Suyoung Seo, Bokyung Son, Minsuk Kong

**Affiliations:** 1 Department of Food Science and Biotechnology, Research Institute of Food and Biotechnology, Seoul National University of Science and Technology, Seoul 01811, Republic of Korea; suyoung4330@snu.ac.kr; 2 Department of Food Biotechnology, Dong-A University, Busan 49315, Republic of Korea; bkson@dau.ac.kr

**Keywords:** *Clostridium perfringens*, bacteriophage, endolysin, cell wall-binding domain, biocontrol

## Abstract

**Background:** *Clostridium perfringens* is a significant cause of food poisoning. Broad-spectrum antibiotics, commonly used to control *C. perfringens*, are becoming less effective due to the rise of antibiotic-resistant strains, necessitating alternative control strategies. **Methods:** A *C. perfringens*-infecting bacteriophage, Dolk21, and its endolysin, PlyDolk21, were isolated and characterized. The lytic activity of PlyDolk21 was assessed in comparison to its catalytic domain alone. Both PlyDolk21 and its cell wall binding domain (CBD) were evaluated in beef and milk for their antimicrobial activity and cell wall binding activity, respectively. **Results:** While phage Dolk21 was specific to certain *C. perfringens* strains, PlyDolk21 exhibited lytic activity against all *C. perfringens* strains tested. The full-length PlyDolk21 showed stronger lytic activity compared to its catalytic domain alone. PlyDolk21_CBD successfully bound to *C. perfringens* in vitro and in foods. Additionally, PlyDolk21 effectively reduced the viable cell counts of *C. perfringens* by 3-log in beef soup and milk samples. **Conclusions:** This study demonstrates that PlyDolk21 and its CBD hold potential as a biocontrol and detection agent targeting *C. perfringens* in various food matrices.

## 1. Introduction

*Clostridium perfringens* is a Gram-positive, rod-shaped, spore-forming anaerobe that causes a wide range of human and veterinary diseases such as gas gangrene and non-foodborne gastrointestinal infections [1,2]. In addition, *C. perfringens* is responsible for two distinct foodborne diseases in humans [3,4]. One is a common form of foodborne illness, typically leading to mild symptoms, which is classic A diarrhea, while the other is Type C necrotic enteritis, a more severe but rare condition [3]. In the U.S., *C. perfringens* is the second most important foodborne pathogen [5,6], and is also responsible for over 4 million infections worldwide annually [7]. Broad-spectrum antibiotics are frequently used to manage *C. perfringens*, but the emergence of antibiotic-resistant strains has reduced the effectiveness of these traditional treatments [8]. Therefore, alternative strategies for controlling this bacterium are crucial.

Bacteriophages and their lytic enzyme, endolysins, have garnered significant attention as a potential solution for combating antibiotic-resistant bacteria [9,10]. During phage infection, endolysin is synthesized in the late stage of phage multiplication, followed by degrading the peptidoglycan layer of host bacteria [11,12]. The use of phages has many advantages including host specificity, self-amplification, and safety [13,14]. However, lysogenic phages could carry the risk of transferring virulent or antibiotic-resistant genes [15]. In contrast, endolysins offer additional benefits over the use of phages, such as broad-spectrum activity, no transduction issues, and the absence of reported bacteria, making them promising candidates for next-generation antimicrobial agents [10]. The structures of endolysins are different between Gram-positive and Gram-negative phages [16]. Endolysins from Gram-negative phages are typically single-domain proteins, lacking a cell wall-binding domain (CBD), because the outer membrane limits access to the peptidoglycan, reducing the need for a cell-binding domain. In contrast, most endolysins from Gram-positive phages consist of more than two domains, mainly an N-terminal enzymatic active domain (EAD) and a C-terminal CBD, which are connected by a flexible linker [17]. Endolysins from *C. perfringens* phages generally exhibit *C. perfringens*-specific lytic activity, with the CBD likely contributing to the species specificity of these endolysins [18]. However, recent X-ray crystal structures and modeling studies of *C. perfringens* endolysins have suggested that the EAD itself may also have species-specific enzymatic activity because the endolysin cleavage site is located near the unique peptide bridges in the *C. perfringens* peptidoglycan [19]. Thus, analysis of each domain is as important as studying full-length endolysin since this approach allows for a better understanding of the specific contributions of both the EAD and CBD to species specificity and lytic activity. Such insights can inform the development of more targeted and effective antimicrobial agents.

There has been a growing number of studies of *C. perfringens*-targeting endolysins, including those with Generally Recognized as Safe (GRAS, GRN000802) [20]-approved status [19,21]. However, while endolysins have been extensively studied in clinical and veterinary settings, research into their application in food systems remains relatively limited [22]. This gap highlights the need for further exploration of endolysins as potential antimicrobial agents in food safety and preservation.

In this study, a novel virulent *C. perfringens* phage Dolk21 was isolated and its biological and genomic characteristics were investigated. An endolysin gene, *plyDolk21*, was identified in the genome of the phage Dolk21. The antimicrobial spectrum and lytic activity of the full-length PlyDolk21 were compared to those of its enzymatically active domain (PlyDolk21_EAD). Predicted CBD (PlyDolk21_CBD) was purified with EGFP and tested for its cell wall-binding ability. In foods, the full-length endolysin was evaluated for its antimicrobial ability against *C. perfringens* and the CBD protein for its binding ability to *C. perfringens*. The results could accelerate developing biocontrol and detection technologies for the effective control of *C. perfringens* foodborne illnesses.

## 2. Results and Discussion

### 2.1. Analysis and Characterization of C. perfringens Phage Dolk21

#### 2.1.1. Lytic Activity and Host Range of Phage Dolk21

A novel bacteriophage, Dolk21, was isolated from a soil sample in Bucheon, Republic of Korea using *C. perfringens* ATCC 13124 as the host strain. To determine the host range of the phage Dolk21, a spotting assay was conducted, with bacterial strains shown in Table 1. The Dolk21 phage was capable of lysing four out of the ten *C. perfringens* strains tested, forming clear plaques. All other Gram-positive and Gram-negative bacterial species exhibited resistance to Dolk21. These findings indicate that the phage Dolk21 has highly restricted host specificity, prompting us to explore alternative approaches, such as utilizing its endolysin, to develop an efficient biocontrol agent capable of targeting multiple strains of *C. perfringens*.

#### 2.1.2. Morphology and Genome Characterization

**Figure 1:**
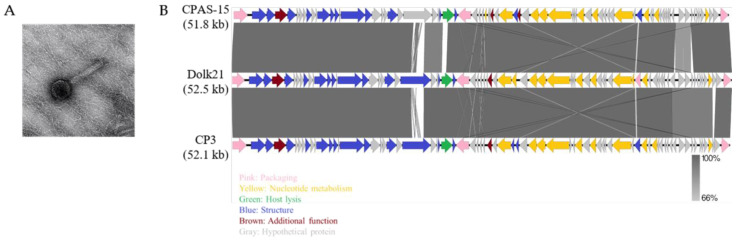
Characterization of bacteriophage Dolk21. (**A**) TEM morphology of Dolk21 and (**B**) genome comparison among three *C. perfringens* phages Dolk21, CPAS-15, and CP3.

The TEM analysis revealed that the Dolk21 phage belongs to the class *Caudoviricetes*. Dolk21 has a thick and contractile tail, indicative of a myovirus-like morphology (Figure 1A). Its icosahedral head is 74.49 ± 3.40 nm (n = 13), and the length of its tail is 155.74 ± 8.24 nm (n = 12). The genome sequence analysis of *C. perfringens* phage Dolk21 indicated that its genome consists of 52,463 base pairs of double-stranded DNA, containing 34% of G+C content The phage’s genome encodes a total of 75 ORFs, of which the functional roles of 31 ORFs were predicted. The functional ORFs were classified into five functional groups of packaging, structure, host lysis, nucleotide metabolism, and additional function (Figure 1B). As there were no lysogeny-related genes found, such as an integrase, a recombinase, or a repressor, this suggested that Dolk21 is likely a virulent phage. BLASTN analysis revealed that the Dolk21 genome shares 97.8% and 98.0% of its DNA sequence identity with *Clostridium* phages CP3 (GenBank Accession No. ASZ76631.1) and CPAS-15 (GenBank Accession No. QGF20128.1), respectively.

### 2.2. Production and Characterization of PlyDolk21

#### 2.2.1. Identification and Expression of the Endolysin PlyDolk21

**Figure 2:**
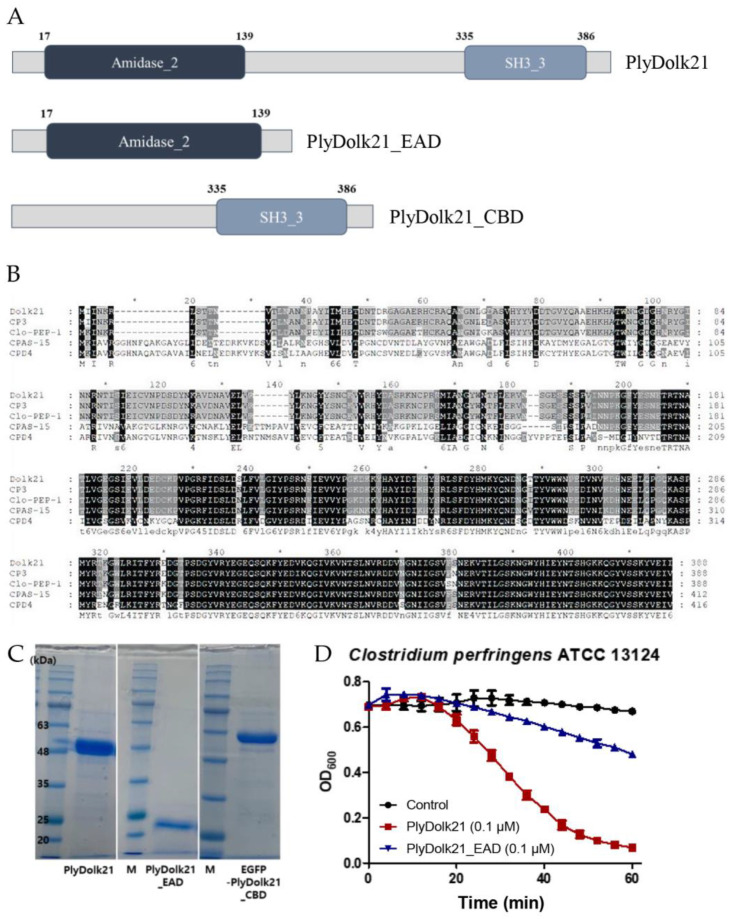
Modular structures and expressions of PlyDolk21 from Dolk21. (**A**) Schematic representation of PlyDolk21, PlyDolk21_EAD, and PlyDolk21_CBD. (**B**) Amino acid sequence alignment of various *C. perfringens* phage endolysins: CP3 phage endolysin, Clo-PEP-1 phage endolysin, CPAS-15 phage endolysin, CPD4 phage endolysin. An asterisk (*) is marked every 10 amino acids. (**C**) SDS-PAGE analysis of purified PlyDolk21, PlyDolk21_EAD, and EGFP-fused PlyDolk21_CBD. M, standard molecular weight marker; PlyDolk21, purified PlyDolk21 fraction; PlyDolk21_EAD, purified PlyDolk21_EAD fraction; PlyDolk21_CBD, purified PlyDolk21_CBD fraction. (**D**) Lytic activities of PlyDolk21 and its EAD.

The putative endolysin gene was identified from the phage Dolk21 genome and designated as *plyDolk21*. Amino acid sequence analysis using BLASTP (https://blast.ncbi.nlm.nih.gov/Blast.cgi?PAGE=Proteins, accessed on 7 January 2025) and InterProScan (version 5.72-103.0) revealed that PlyDolk21 consists of two functional domains, an N-terminal amidase_2 domain as the EAD and a C-terminal SH3_3 domain as the CBD (Figure 2A). The segment between these two domains did not match any known functional domain in the current databases. Further experimental or computational studies would be necessary to assign a potential function to this region. BLASTP analysis showed several proteins homologous to PlyDolk21, but most of them have not yet been studied. The amino acid sequence of the PlyDolk21 was aligned with those of endolysins from *C. perfringens* phages CP3, Clo-PEP-1 (GenBank Accession No. APQ41998.1), CPAS-15, and CPD4 (GenBank Accession No. MK017819.1), which exhibit high similarity to PlyDolk21 (Figure 2B). Notably, the sequence of the putative CBD of PlyDolk21 is highly homologous to that of the phage CPAS-15 endolysin, which was experimentally confirmed to have cell wall-binding activity, while the EAD differs significantly. Based on this information, the PlyDolk21 and each domain were cloned and expressed in *E. coli* with an N-terminal His-tag. A single band of each purified protein was identified on sodium dodecyl sulfate polyacrylamide gel electrophoresis (SDS-PAGE), and each band corresponded to the expected molecular mass of the respective protein (Figure 2C).

#### 2.2.2. Antimicrobial Activity of PlyDolk21 and PlyDolk21_EAD

The antimicrobial activity of PlyDolk21 and PlyDolk21_EAD was evaluated by a turbidity reduction assay. The treatment of 0.1 μM of PlyDolk21 and PlyDolk21_EAD dropped the turbidity of the *C. perfringens* cell suspension by 85% and 30%, respectively, indicating that CBD was necessary for PlyDolk21 to have its maximal lytic activity (Figure 2D). This phenomenon was particularly observed when endolysins targeted their natural host bacteria. In a previous study, the lytic activity of LysPBC1 and its enzymatically active domain (LysPBC1_EAD) was compared against both target and non-target bacteria. The findings showed that the C-terminal domain of LysPBC1 was required for full activity against its natural target, but not for non-target bacteria [23].

The antimicrobial spectral analysis showed that PlyDolk21 and PlyDolk21_EAD inhibited the growth of all tested strains of *C. perfringens*, *B*. *subtilis*, *P*. *aeruginosa*, and *C. sakazakii*, demonstrating a much broader lytic spectrum compared to phage Dolk21 (Table 1). It should be noted that PlyDolk21 and PlyDolk21_EAD showed antimicrobial activity against *P. aeruginosa* and *C. sakazakii* without the addition of outer membrane permeabilizers. This lytic activity may be attributed to the attached His-tag, which likely enhanced interactions with the highly negatively charged outer membrane of *P. aeruginosa* and *C. sakazakii* [24]. In particular, the membrane lipid A of *P. aeruginosa* was modified with 4-amino-4-deoxy-L-arabinose (L-Ara4N) or phosphoethanolamine, further increasing its negative charge and potentially facilitating stronger electrostatic interactions with the His-tag of the proteins tested [25]. Similarly, *C. sakazakii* possesses a negatively charged outer membrane with lipid A modifications like L-Ara4N or phosphoethanolamine [26]. The turbidity reduction assay using PlyDolk21_EAD without the His-tag exhibited abolished antimicrobial activity (Figure S1), further emphasizing the critical role of the His-tag in facilitating interactions with the negatively charged outer membrane of *P. aeruginosa* and C. sakazakii. However, the exact mechanisms by which these endolysins permeate the outer membrane remain unclear. Notably, PlyDolk21_EAD also exhibited lytic activity against *L*. *monocytogenes* and *Geobacillus stearothermophilus*. It is known that the cell wall-binding domain of endolysins can inhibit lytic activity when not bound to its target [27], which may explain why the antimicrobial spectrum of the PlyDolk21_EAD appeared broader than that of the full-length endolysin, PlyDolk21. In this case, the presence or absence of the His-tag could not have significantly affected the activity of PlyDolk21_EAD. Indeed, we observed that the lytic rate of PlyDolk21_EAD against *G. stearothermophilus* was even faster in the absence of the His-tag (Figure S1), suggesting that its activity against *L. monocytogenes* and *Geobacillus* may not have strongly relied on the presence of the His-tag.

#### 2.2.3. Stability of PlyDolk21 Under Various Stress Conditions

**Figure 3:**
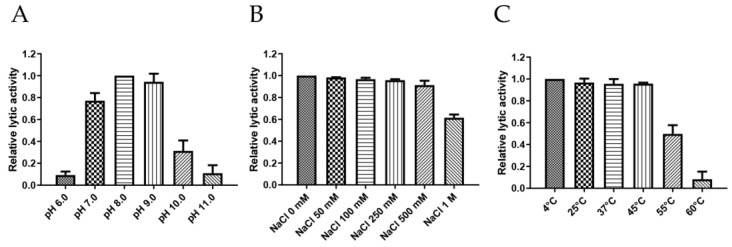
Stability of PlyDolk21 under various stress conditions was tested. Effects of (**A**) pH, (**B**) NaCl, and (**C**) temperature on the lytic activity of PlyDolk21 against *C. perfringens* ATCC 13124 cells. The means of triplicate experiments are represented in each column, and error bars indicate the standard deviation.

Endolysin should maintain stable activity under various stress conditions, including pH, temperature, and NaCl concentrations, to ensure its broad applicability [28]. To this end, the stability of PlyDolk21 was tested under a broad range of pH (6.0 to 11.0), temperature (4 °C to 60 °C), and NaCl concentrations (0 mM to 1000 mM) (Figure 3). PlyDolk21 showed maximal lytic activity between pH 7.0 and 9.0, but its activity diminished under acidic conditions (pH < 6.0) (Figure 3A). PlyDolk21 was relatively stable across all tested NaCl concentrations, maintaining high levels of lytic activity (Figure 3B). When tested under varying temperatures, its lytic activity remained stable between 4 °C and 45 °C, but gradually declined at 55 °C (Figure 3C).

### 2.3. Isolation and Binding Activity Analysis of PlyDolk21_CBD

**Figure 4:**
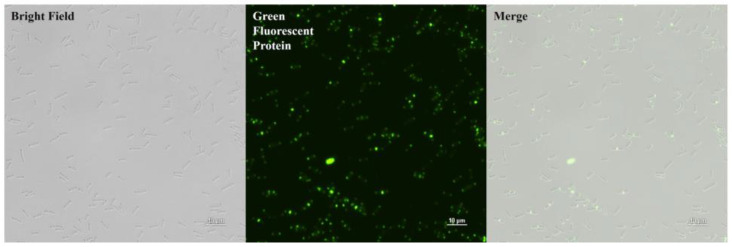
Binding activity of PlyDolk21_CBD to cell wall of *C. perfringens* in KH_2_PO_4_-NaOH buffer. Representative cell images show *C. perfringens* cells with PlyDolk21_CBD fused with EGFP. Panels from left to right show bright field, PlyDolk21_CBD with EGFP, and merged image.

PlyDolk21_CBD was purified as shown in Figure 2C. The specific bacterial binding activity of the putative PlyDolk21_CBD was identified with an enhanced green fluorescent protein (EGFP), demonstrating that it primarily binds to the septal region and pole of the cells (Figure 4). The septal region undergoes active cell wall remodeling during cell division. Since amidases are involved in peptidoglycan hydrolysis in the septal region, its CBD could have high affinity to this region [29,30]. All tested *C. perfringens* strains were detected by EGFP_PlyDolk21_CBD, but not other *Clostridium* species or other Gram-positive and Gram-negative bacteria (Table 1 and Figure S2). These findings suggest that PlyDolk21_CBD could serve as a valuable material for the development of *C. perfringens* detection tools.

### 2.4. Food Applications

#### 2.4.1. Bacterial Control of PlyDolk21 in Food Samples

**Figure 5:**
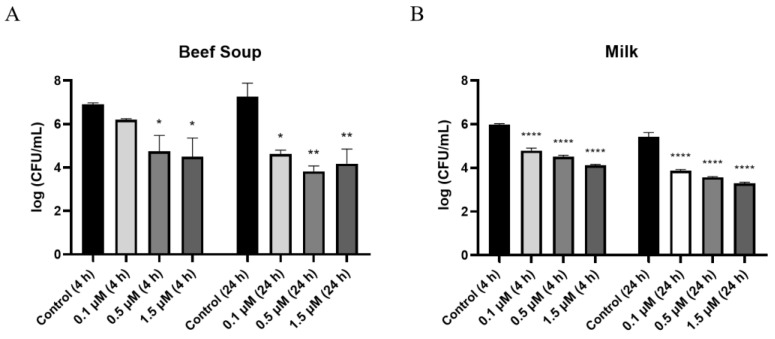
Food applications of PlyDolk21 in (**A**) beef soup and (**B**) milk contaminated with *C. perfringens* ATCC 13124. Control, *C. perfringens*-contaminated food sample without PlyDolk21 (black); 0.1 μM, 0.5 μM, and 1.5 μM PlyDolk21-treated *C. perfringens*-contaminated beef soup or milk samples. PlyDolk21 was incubated with *C. perfringens* contaminated food samples for 4 or 24 h. Error bars present the standard deviations of three replicates. Asterisks indicate significant differences (*, *p* < 0.05; **, *p* < 0.01; ****, *p* < 0.0001).

As commercial milk and beef are often reported to be contaminated with *C. perfringens* [31], sterilized milk and beef soup were chosen to evaluate the inhibitory activity of PlyDolk21 against *C. perfringens* in a food environment. In beef soup, treatment with 0.1 μM PlyDolk21 resulted in a 1-log reduction in CFUs after 4 h of incubation and a 3-log reduction after 24 h. (Figure 5A). When the concentration increased to 0.5 μM, a 4-log reduction in CFUs was observed after 24 h of incubation (Figure 5A). In sterilized milk, treatment with 1.5 μM PlyDolk21 resulted in a 3-log reduction in *C. perfringens* counts after 24 h of incubation (Figure 5B). These findings suggest that PlyDolk21 could be utilized as a natural food preservative to control *C. perfringens* in milk and beef soup. There are a few studies that explore the potential of using endolysins in foods to inhibit *C. perfringens*. For instance, LysCP28 from *C. perfringens* phage vB_CpeS_BG3P was evaluated for its efficacy in reducing *C. perfringens* in duck meat [32]. Additionally, the phage endolysin cpp-lys was applied to lettuce, highlighting the promising role of endolysins as biocontrol agents in various food systems [33].

#### 2.4.2. Cell Wall-Binding Ability of PlyDolk21_CBD in Foods

**Figure 6:**
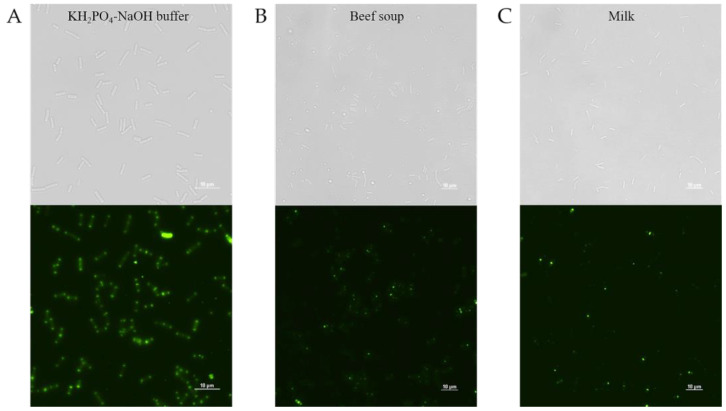
Food applications of PlyDolk21_CBD in (**A**) KH_2_PO_4_-NaOH buffer, (**B**) beef soup, and (**C**) milk contaminated with *C. perfringens* ATCC 13124. Representative cell images show *C. perfringens* cells with PlyDolk21_CBD fused with EGFP. Top and bottom panels show bright field and PlyDolk21_CBD fused with EGFP, respectively.

To evaluate the detection ability of PlyDolk21_CBD in foods, PlyDolk21_CBD fused with EGFP was added to a sterilized milk and beef soup artificially contaminated with *C. perfringens*. Given the presence of various components in food, CBD-bound cells were monitored after thorough washing. Although its binding activity was reduced by approximately 43.2% in milk and 38% in beef soup compared to that in the buffer, the EGFP-fused PlyDolk21_CBD successfully bound to *C. perfringens* within 5 min (Figure 6 and Figure S3). These findings suggest that the CBD protein may be useful for the rapid detection of specific pathogens in food [34,35].

## 3. Materials and Methods

### 3.1. Bacterial Strains and Growth Conditions

*C. perfringens* ATCC 13124 was used as a host strain for the isolation and propagation of the phage Dolk21. All tested strains are listed in Table 1. *Clostridium* strains and *L. monocytogenes* ATCC 15313 were grown in Brain Heart Infusion (BHI) broth at 37 °C under anaerobic conditions. *B. subtilis* ATCC 23857, *S. aureus* Newman, and *C. sakazakii* ATCC 29544 were grown in Tryptic Soy (TS) broth at 37 °C under shaking and *Geobacillus stearothermophilus* ATCC 10149 was grown in the same medium at 50 °C under shaking. *B. cereus* ATCC 10987 and the rest of the Gram-negative bacteria were grown in Luria–Bertani (LB) broth at 37 °C. *E. coli* BL21 was grown in LB broth at 37 °C and used for expression of the recombinant PlyDolk21 and its domains.

### 3.2. Isolation and Propagation of Bacteriophage Dolk21

A soil sample was collected from Bucheon, Republic of Korea. Phage isolation was performed as previously described with some modifications [23]. A 10 g sample was homogenized with 10 mL 2× BHI broth and 10 mL mixture was incubated with 400 μL cultured bacteria, 5 mM MgCl_2_, and 5 mM CaCl_2_. After an overnight anaerobic incubation at 37 °C, the culture was centrifugated (10,000× *g* at 4 °C for 10 min) and the supernatant was filtered using a 0.45 μm pore size filter (Sartorius AG, Göttingen, Germany). The presence of phages was confirmed using a plaque-forming assay with molten 0.7% DW soft agar inoculated with *C. perfringens* ATCC 13124 overnight cultures. After incubating at 37 °C overnight, a single plaque was picked with a sterile pipette tip and eluted in 1 mL of SM buffer (50 mM Tris-HCl [pH 7.5], 100 mM NaCl, and 10 mM MgCl_2_). These plaque isolation and elution steps were repeated five times to purify the single phage. Isolated phages were amplified through serial propagation and concentrated via polyethylene glycol (PEG) precipitation, followed by CsCl density gradient ultracentrifugation (78,500× *g* at 4 °C for 2 h) [36]. The concentrated phages were dialyzed with SM buffer for 2 h, with the buffer being changed two times during the dialysis.

### 3.3. Morphological Analysis by TEM

Purified Dolk21 (1.2 × 10^10^ PFU/mL) was placed on carbon-coated copper grids and negatively stained with 2% aqueous uranyl acetate (pH 4.0) by pipetting 10 times [23]. Dolk21 was visualized by energy-filtering transmission electron microscopy (EF-TEM, Thermo Fisher Scientific, Waltham, MA, USA) at 120 kV. Images were scanned at NICEM at Seoul National University (Seoul, Republic of Korea).

### 3.4. DNA Purification and Whole Genome Sequencing of Bacteriophage Dolk21

The Dolk21 genomic DNA extraction was carried out as previously described [36]. The purified genomic DNA of Dolk21 was sequenced using the Illumina Miseq platform (San Diego, CA, USA) and assembled with SPAdes v.3.15.2 (Sanigen, Anyang, Republic of Korea).

### 3.5. Bioinformatic Analysis

Functions of the predicted ORFs were confirmed and annotated using BLASTP and InterProScan [37] programs. The genome annotation identified an ORF encoding the endolysin PlyDolk21, along with its EAD and CBD. The primary protein structure of PlyDolk21 was compared with the endolysins of *Clostridium* phages (endolysin of phage CP3, NCBI Accession ID ASZ76631.1; endolysin of phage Clo-PEP-1, NCBI Accession ID APQ41998.1; endolysin of phage CPAS-15, NCBI Accession ID QGF20128.1; endolysin of phage CPD4, NCBI Accession ID KQC91385.1) using ClustalX2.1 and Genedoc v.2.6. To locate the EAD and CBD domains, secondary protein structures of PlyDolk21 were predicted using the NPS@ server (https://npsa.lyon.inserm.fr/cgi-bin/npsa_automat.pl?page=/NPSA/npsa_sopma.html, accessed on 7 January 2025) and PSIPRED v.4.02 tool, and tertiary protein structures of PlyDolk21 were predicted using the Phyre v.2.0 server and PyMOL v.3.0 program.

### 3.6. Cloning, Expression, and Purification

The endolysin gene (*plyDolk21*) was amplified from the genomic DNA of the bacteriophage Dolk21 by PCR using primers fPlyDolk21_BamHI (5′-GCG GGATCC ATGATAATCAATAAAAGATTAAGTACTACTAATGTTACCTTAAAC-3′) and rPlyDolk21_HindIII (5′-GCG AAGCTT CTAAATTATCTCTACATATTTTGAACTTACATAACCTTGC-3′). Additionally, the reverse primer rPlyDolk21_EAD_HindIII (5′-GCG AAGCTT CTATGAATTTACTCTTTCTAGGAATGTATTCCAGTAACC-3′) was designed for its EAD domain, and the forward primer fPlyDolk21_CBD_ BamHI (5′-GCG GGATCC GGAGAGAGTTCAAGCTCACCAGTAATTAATAATC-3′) was also designed for its CBD domain. The PCR products of *plyDolk21* and *plyDolk21_EAD* were cloned into pET28a, which has an N-terminal hexahistidine (His6)-tag sequence. To check the binding to the cell wall with fluorescence, the enhanced green fluorescent protein (EGFP) gene was inserted in front of PlyDolk21_CBD using the NheI and BamHI restriction sites. Plasmids with each insert were transformed into a competent *E. coli* BL21 (DE3) cell. Each protein was overexpressed and purified following the methods described in a previous study [23]. A Thrombin CleanCleave Kit (Sigma-Aldrich, St. Louis, MO, USA) was used for N-terminus His6-tag cleavage. A total of 100 μL of washed thrombin–agarose resin was resuspended in 100 μL of 10X cleavage buffer (500 mM Tris-HCl, pH 8.0, 100 mM CaCl_2_), and 800 μg of purified PlyDolk21_EAD was added. The mixture was subsequently allowed to react at room temperature for 3 h, during which it was stirred gently. The cleavage of the His6-tag was confirmed using SDS-PAGE analysis (Figure S1).

### 3.7. Antimicrobial Spectrum of the Phages Dolk21, PlyDolk21, and PlyDolk21_EAD

The host range of the phage Dolk21 was confirmed, with the strains listed in Table 1. After incubation overnight, 100 μL of each strain was added to 5 mL of 0.7% molten soft agar and overlaid on a suitable agar plate. Then, 10 μL of each serially diluted phage suspension was spotted on the plate and incubated at 37 °C overnight. The antimicrobial spectra of PlyDolk21 and PlyDolk21_EAD were evaluated by turbidity reduction assay [23]. Bacterial cells were early exponentially grown and resuspended with reaction buffer (20 mM Tris-Cl, pH 8.0) to adjust the OD_600_ to approximately 0.6. The purified protein was added to a final concentration of 0.4 μM, and the inoculated cell resuspension was cultured at 25 °C. The OD_600_ values were monitored for 30 min with 1 min intervals at room temperature. The relative lytic activity (%) of PlyDolk21 and PlyDolk21_EAD was calculated using the Equation (1) presented below.

(1) $$\left[\frac{\left(\mathrm{final}\;{OD}_{600}\;\mathrm{of}\mathrm{treatment}-\;\mathrm{final}\;{OD}_{600}\mathrm{of}\mathrm{control}\right)}{\mathrm{initial}{OD}_{600}}\times 100\;(\%)\right]$$

### 3.8. Stability Under Various Conditions

To evaluate the effect of pH on PlyDolk21, endolysin was added to *C. perfringens* ATCC 13124 cells suspended with a universal pH buffer. The universal buffer (0.04 M H_3_PO_4_, 0.04 M CH_3_COOH, 0.04 M H_3_BO_3_) was adjusted to different pH values, between 6.0 to 11.0, using 5 M NaOH. The effect of temperature on PlyDolk21 was tested at different temperatures (4–60 °C). The endolysin was incubated at each temperature for 20 min and added to cells suspended with reaction buffer. The influence of NaCl on PlyDolk21 was tested with reaction buffer containing 0 to 1000 mM NaCl. In each case, PlyDolk21 was treated to a final concentration of 0.4 μM, and the inoculated cell resuspension (10^6^ CFU/mL) was incubated at 25 °C. The OD_600_ values were monitored for 30 min at 1 min intervals. The lytic activity under each stress condition was relatively measured based on Equation (2).

(2) $$\left[\frac{{\left(\left|\mathrm{initial}{OD}_{600}-40\min{OD}_{600}\right|\right)}_{\mathrm{Each}\mathrm{condition}\mathrm{of}\mathrm{treatment}}}{{\left(\left|\mathrm{initial}{OD}_{600}-40\min{OD}_{600}\right|\right)}_{\mathrm{Condition}\mathrm{of}\mathrm{maximal}\mathrm{activity}}}\times 100\;(\%)\right]$$

### 3.9. Food Applications of PlyDolk21

Milk and beef soup, which are frequently found with *C. perfringens*, were selected as model food samples. The antimicrobial activity in each food sample was tested as previously described [38]. Each sample artificially contaminated with *C. perfringens* ATCC 13124 (10^6^ CFU/mL) was preincubated at 25 °C for 30 min. Subsequently, purified PlyDolk21 at concentrations of 0.1 μM, 0.5 μM, and 1.5 μM was added to the beef soup and milk. The mixture was incubated at 25 °C for 4 and 24 h. The antimicrobial activity of endolysin was determined by calculating the viable cell count, comparing between the control and test groups. All experiments were repeated three times. One-way analysis of variance (ANOVA) and the Tukey’s multiple comparison test were used for statistical analysis.

### 3.10. Cell Wall-Binding Assay with Fluorescence Microscopy of PlyDolk21_CBD

The results of the amino acid sequence alignment showed that the CBD region of PlyDolk21 has high similarity to that of *C. perfringens* phage endolysin LysCPAS15 [34]. Based on this finding, the CBD part of the gene *plyDolk21* was cloned in fusion with a gene encoding EGFP and expressed in *E. coli*. To confirm the binding activity of PlyDolk21_CBD, 100 μL of purified protein at a final concentration of 1 μM was added to 100 μL of *C. perfringens* cells (10^6^ CFU/mL) resuspended in KH_2_PO_4_-NaOH buffer (pH 7.0), and the mixture was incubated at RT for 5 min. Subsequently, cells were collected by centrifugation (16,000 × *g* for 1 min) and washed twice with KH_2_PO_4_-NaOH buffer (pH 7.0). Resuspended pellet with 10 μL KH_2_PO_4_-NaOH buffer (pH 7.0) was dropped on a slide glass and covered with an agarose pad (0.1 g of agarose powder (TransGen) + 13 mL of distilled water). An inverted microscope (ECLIPSE Ti2-E, Nikon, Tokyo, Japan) was used in this assay (DIA: 17.1, Intensilight ND 2, 1 s Auto exposure, 1.0× Analog Gain). For quantification, washed cells were resuspended in 200 μL of PBS buffer and their fluorescence was measured using a SpectraMax i3x plate reader (Molecular Devices, San Jose, CA, USA) with excitation at 485 nm and emission at 520 nm.

### 3.11. Food Applications of EGFP-Fused PlyDolk21_CBD

The ability of the endolysin PlyDolk21 to control *C. perfringens* ATCC 13124 was demonstrated in the milk and beef soup. To evaluate the detection ability of PlyDolk21_CBD in each food, a cell wall-binding assay was conducted. Using the same procedure as for the endolysin assay, prepared *C. perfringens* ATCC 13124 cells were added to sterilized milk and beef soup. To remove the unbound CBD protein and other components in food samples such as fat, the mixtures were washed twice in the beef soup sample and four times in the milk sample using sterilized PBS buffer. The binding ability was confirmed as described above.

### 3.12. Nucleotide Sequence Accession Number

The complete genome sequence of *C. perfringens* phage Dolk21 is available in the GenBank database under Accession No. OP730325.

## 4. Conclusions

In this study, the novel bacteriophage Dolk21 was isolated from a soil sample using *C. perfringens* ATCC 13124 as the host strain. The phage demonstrated highly restricted host specificity, which prompted further investigation into using its endolysin as an alternative. PlyDolk21, the endolysin encoded by the phage Dolk21, exhibited strong lytic activity against a broader range of *C. perfringens* strains compared to the phage itself, showing potential as an efficient biocontrol agent in food safety. Its stability under various environmental conditions such as pH, temperature, and NaCl concentration supports its use in foods. Furthermore, the rapid detection ability of its CBD provides an additional advantage for *C. perfringens* identification. PlyDolk21 shows significant promise as a natural and safe alternative for controlling *C. perfringens* contamination in food products. A future optimization study of PlyDlk21 could enhance its efficacy and expand its applications in the food industry.

## Figures and Tables

**Table 1:** Host range of phage Dolk21, lytic activity of PlyDolk21 and its enzymatic active domain PlyDolk21_EAD, and cell wall-binding activity of PlyDolk21_CBD.

Species	Strain No. ^a^	Dolk21 ^b^	PlyDolk21 ^c^	PlyDolk21_EAD ^c^	PlyDolk21_CBD ^d^
*C. perfringens*	2	+	+++	++	+
*C. perfringens*	24	+	++	+	+
*C. perfringens*	2585	−	+++	+	+
*C. perfringens*	2589	+	+++	+	+
*C. perfringens*	ATCC 3624	−	+++	+	+
*C. perfringens*	ATCC 13124	+	+++	++	+
*C. perfringens*	NCCP 15911	−	++	+	+
*C. perfringens*	H3	−	++	+	+
*C. perfringens*	H9	−	++	+	+
*C. perfringens*	FD1	−	+++	++	+
**Other Gram-positive**					
*Bacillus cereus*	ATCC 10987	−	−	−	−
*Bacillus subtilis*	ATCC 23857	−	+	++	−
*Staphylococcus* *aureus*	Newman	−	−	−	−
*Listeria* *monocytogenes*	ATCC 15313	−	−	+	−
*Geobacillus* *stearothermophilus*	ATCC 10149	−	−	++	−
*Levilactobacillus brevis*	ATCC 11433	−	−	−	−
**Gram-negative**					−
*E. coli* O157:H7	ATCC 35150	−	−	−	−
*Salmonella* Typhimurium	LT2	−	−	−	−
*Pseudomonas* *aeruginosa*	PAO1	−	+	+	−
*Cronobacter* *sakazakii*	ATCC 29544	−	+	+	−

^a^ ATCC, American Type Culture Collection; NCCP, National Culture Collection for Pathogens. ^b^ +, activity of dotting assay; −, no activity. ^c^ −, 0–10%; +, 11–40%; ++, 41–70%; +++, 71–100%. ^d^ +, activity of cell wall-binding assay; −, no activity.

## Data Availability

The original contributions presented in this study are included in this article, and further inquiries can be directed to the corresponding author.

## Supplementary Materials

The following supporting information can be downloaded at: https://www.mdpi.com/article/10.3390/antibiotics14010081/s1, Figure S1. Antimicrobial activity of PlyDolk21_EAD and His-tag removed PlyDolk21_EAD against various bacterial strains. (A) SDS-PAGE analysis of purified PlyDolk21_EAD and His-tag removed PlyDolk21_EAD. Lane M: protein marker; Lane 1: PlyDolk21_EAD; Lane 2: His-tag removed PlyDolk21_EAD. Lytic activities of PlyDolk21 and His-tag removed PlyDolk21_EAD against *Pseudomonas aeruginosa* ATCC 15692 (B), *Cronobacter sakazakii* ATCC 29544 (C), *Listeria monocytogenes* ATCC 15313 (D), and *Geobacillus stearothermophilus* ATCC 10149 (E). Closed circles represent the control group (no treatment), open triangles represent His-tag removed PlyDolk21_EAD, and closed triangles represent PlyDolk21_EAD. Data represent the mean ± standard deviation of three independent experiments. Figure S2. EGFP_PlyDolk21_CBD did not bind to *Geobacillus stearothermophilus* cells, which were used as a representative negative control. Panels from left to right show bright field and PlyDolk21_CBD with EGFP. Figure S3. The binding activity of EGFP-fused PlyDolk21_CBD in buffer (KH₂PO₄-NaOH, pH 7.0), milk, and beef soup. The activity is expressed as relative fluorescence intensity (RFI) normalized by OD₆₀₀. Error bars present the standard deviations of three replicates. The asterisks indicate significant differences (***, *p* < 0.001).
