# Supplementary material for: Characterization of Clostridium perfringens Phage Endolysin PlyDolk21
Source: Antibiotics (Basel). 2025 Jan 13;14(1):81. doi: 10.3390/antibiotics14010081 (PMC11762992; doi:10.3390/antibiotics14010081)
Supplement: Supplementary file 1 [file antibiotics-14-00081-s001.zip › antibiotics-3303500-supplementary.pdf]

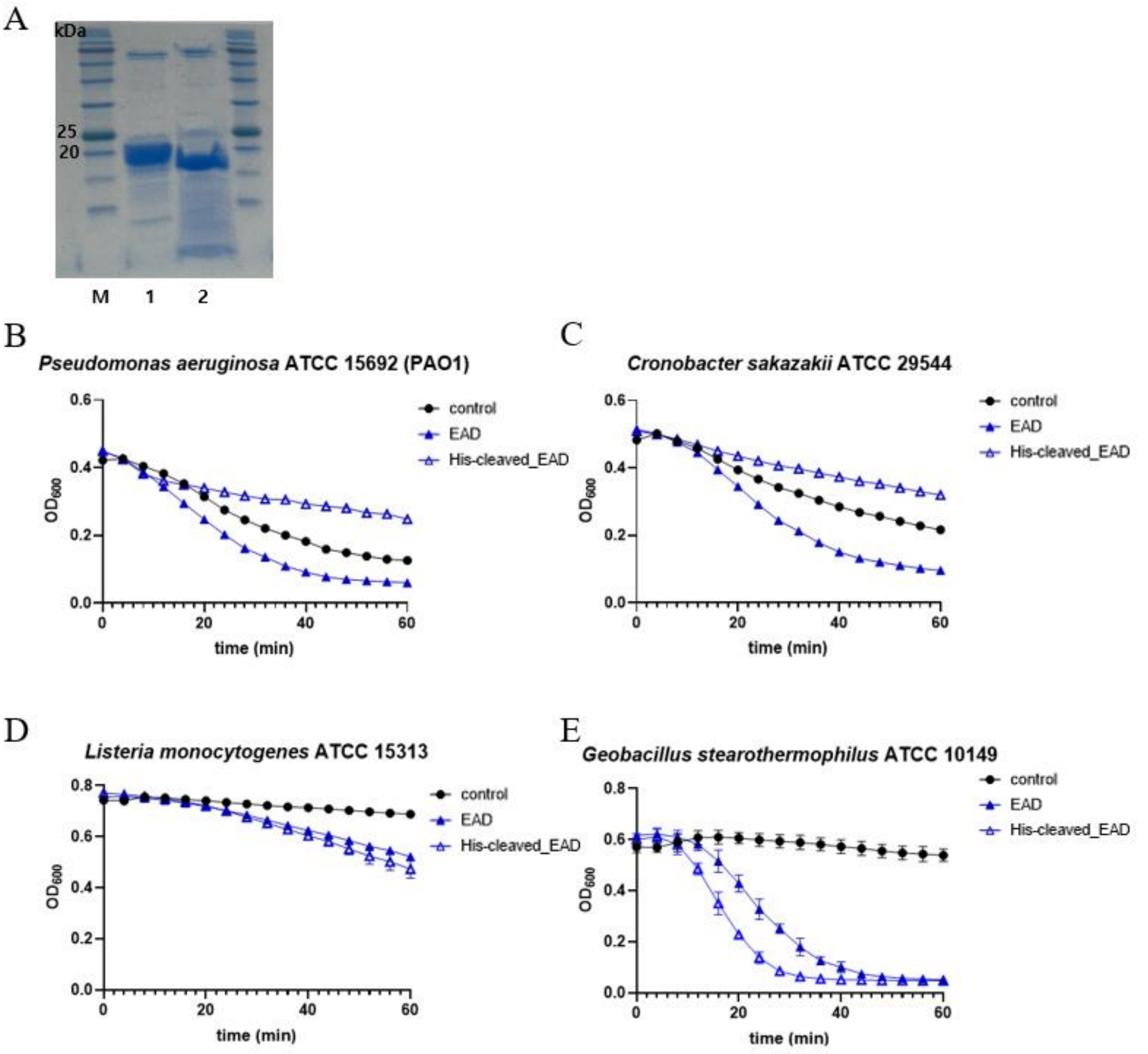

Figure S1. Antimicrobial activity of PlyDolk21_EAD and His-tag removed PlyDolk21_EAD against various bacterial strains. (A) SDS-PAGE analysis of purified PlyDolk21_EAD and His-tag removed PlyDolk21_EAD. Lane M: protein marker; Lane 1: PlyDolk21_EAD; Lane 2: His-tag removed PlyDolk21_EAD. Lytic activities of PlyDolk21 and His-tag removed PlyDolk21_EAD against *Pseudomonas aeruginosa* ATCC 15692 (B), *Cronobacter sakazakii* ATCC 29544 (C), *Listeria monocytogenes* ATCC 15313 (D), and *Geobacillus stearothermophilus* ATCC 10149 (E). Closed circles represent the control group (no treatment), open triangles represent His-tag removed PlyDolk21_EAD, and closed triangles represent PlyDolk21_EAD. Data represent the mean ± standard deviation of three independent experiments.

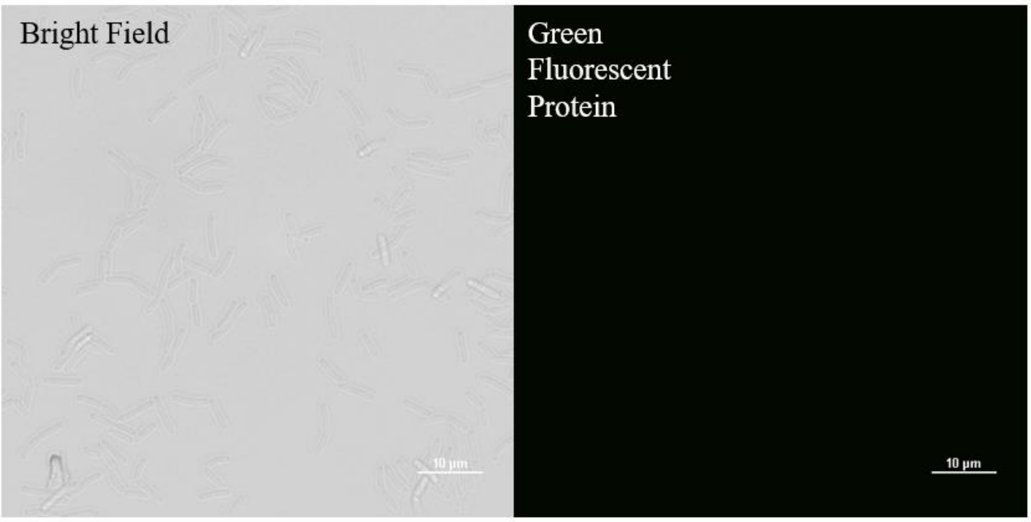

Figure S2. EGFP_PlyDolk21_CBD did not bind to *Geobacillus stearothermophilus* cells, which were used as a representative negative control. Panels from left to right show bright field and PlyDolk21_CBD with EGFP.

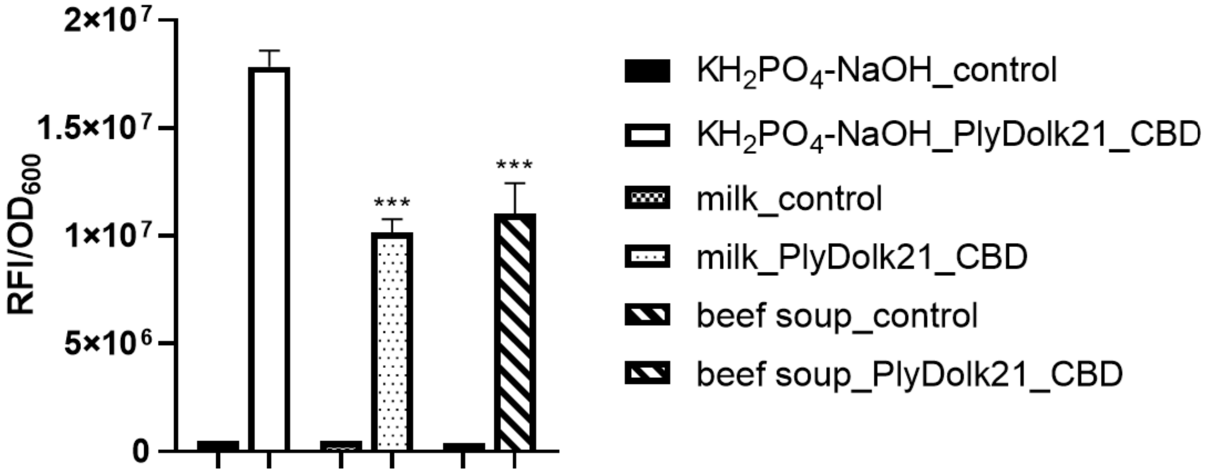

Figure S3. The binding activity of EGFP-fused PlyDolk21_CBD in buffer (KH_2PO_4-NaOH, pH 7.0), milk, and beef soup. The activity is expressed as relative fluorescence intensity (RFI) normalized by OD_{600}. Error bars present the standard deviations of three replicates. The asterisks indicate significant differences (***, $p < 0.001$).
